# Substrate Effect on the Contribution of Ammonium and Urea to Marine Nitrification and Nitrous Oxide Production

**DOI:** 10.1111/1462-2920.70187

**Published:** 2025-10-06

**Authors:** Weiyi Tang, Catherine Hexter, Rongbo Dai, Samantha G. Fortin, John C. Tracey, Naomi Intrator, Moriah A. Kunes, Xianhui S. Wan, Amal Jayakumar, Dalin Shi, Bess B. Ward

**Affiliations:** ^1^ Department of Geosciences Princeton University Princeton New Jersey USA; ^2^ College of Marine Science, University of South Florida St Petersburg Florida USA; ^3^ State Key Laboratory of Marine Environmental Science College of Ocean and Earth Sciences, Xiamen University Xiamen China

**Keywords:** ammonia oxidation, ammonia oxidizers, ammonia‐oxidising archaea, nitrification, nitrous oxide, substrate concentration, substrate ratio, urea oxidation

## Abstract

Nitrification (microbial oxidation of ammonia to nitrite and nitrate) controls nitrogen speciation and is the main source of nitrous oxide (N_2_O) in the ocean. It was recently shown that the most abundant marine ammonia oxidizers, the ammonia‐oxidising archaea (AOA), are also capable of oxidising urea, providing a previously ignored source of nitrite. Here, we show that the relative magnitude of urea and ammonia oxidation rates, and the relative rates of N_2_O production from the two substrates, is correlated with the ratio of the substrate concentrations. By examining all reported measurements of urea and ammonium concentrations and the paired urea and ammonia oxidation rates, we show that this relationship likely holds across the global ocean. Examination of newly acquired and previously published metagenomic data shows that the fraction of AOA with the genetic capability for urea oxidation increases with the urea:ammonium ratio, rather than depending on the urea or ammonium concentration alone. These results corroborate the correlation between substrate ratios and oxidation rate ratios, and extend it to N_2_O production. This may help explain the distribution of nitrification rates and N_2_O production in the ocean.

## Graphical abstract

The urea to ammonium concentration ratio is a good predictor of the relative importance of urea and ammonium to nitrite and N_2_O production in the ocean. In addition, the urea to ammonium ratio may be a key factor in shaping the genetic capability of marine ammonia‐oxidising archaea for urea utilisation.

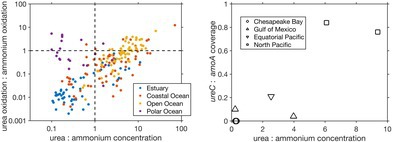

## 1 Introduction

Nitrification plays a key role in the global nitrogen (N) cycle by oxidising ammonia (NH_3_) to nitrite (NO_2_^−^) and further to nitrate (NO_3_^−^) (Ward 2008; Norton and Stark 2011). Nitrification controls the relative availability of inorganic nitrogen compounds that serve as the N source for primary production. Additionally, nitrification consumes oxygen and produces a powerful greenhouse gas—nitrous oxide (N_2_O) (Yool et al. 2007; Santoro et al. 2011). The first step of nitrification, ammonium oxidation (we use ammonium to represent both ammonia and ammonium), is mainly carried out by ammonia‐oxidising archaea and bacteria (AOA and AOB), with AOA dominating in the marine environment (Beman et al. 2012; Tang et al. 2023).

Genomic analysis and culture experiments have shown that some AOA can utilise urea, in addition to ammonium, as an energy, nitrogen and carbon source (Alonso‐Saez et al. 2012; Bayer et al. 2016; Carini et al. 2018; Qin et al. 2024). Urea can also be decomposed into ammonium by other microbes and subsequently utilised by AOA (Koch et al. 2015). Urea concentrations can be comparable to or even higher than ammonium concentrations in some areas of the open ocean (Harrison et al. 1985; Painter et al. 2008). Urea is mainly produced during organic matter remineralization and zooplankton excretion, and its concentration in the ocean is also influenced by human activities (Solomon et al. 2010; Sipler and Bronk 2015). An increasing urea input from anthropogenic sources such as fertiliser applications into estuarine and coastal environments contributes to eutrophication (Glibert et al. 2006), which potentially affects the abundance and activities of ammonia oxidizers.

Urea oxidation has recently been observed in marine environments, suggesting urea is a previously overlooked source of nitrite and N_2_O. For example, urea oxidation rates were comparable to ammonium oxidation rates in the Northwestern Pacific (Wan et al. 2021) and in the Southern California Bight (Laperriere et al. 2020), representing a substantial fraction of the nitrification flux in the oligotrophic ocean (Wan et al. 2024). However, much smaller rates of urea oxidation compared to ammonium oxidation were found in coastal waters near Georgia (United States) (Tolar et al. 2017; Damashek et al. 2018), in the northern Gulf of Mexico (Kitzinger et al. 2018), in Jiulong River Estuary in southeastern China (Tang, Xu, et al. 2022), and in Chesapeake Bay (Tang, Tracey, et al. 2022). The spatial variation in the relative contribution of urea oxidation and ammonium oxidation to nitrite production may be affected by variations in the relative concentrations of urea and ammonium (Wan et al. 2024). Ammonium oxidation is also the major N_2_O production process in the ocean (Santoro et al. 2011; Freing et al. 2012; Ji et al. 2018). However, only one study has directly measured N_2_O production from urea in the marine environment (Tang, Tracey, et al. 2022), leaving the contribution of urea oxidation to N_2_O production largely unknown.

Previous studies have reported a substrate‐dependent biogeographic distribution of ammonium oxidation and urea oxidation across marine environments (e.g., the relationship between substrate ratio and rate ratio of urea oxidation to ammonium oxidation in Wan et al. (2024)). This study aims to assess the importance of ammonium and urea in nitrite and N_2_O production and examine the relationship between substrates and the distributions of ammonium oxidation and urea oxidation in the global ocean. We conducted field measurements of ammonium oxidation, urea oxidation, and their associated N_2_O production in Chesapeake Bay, the largest estuary in the United States, and compared them to a compiled global dataset of ammonium oxidation and urea oxidation rates. Molecular analysis of microbial communities in representative marine systems was performed to determine the genetic capability of AOA to utilise urea in these areas. In the end, a correlation framework was developed to evaluate where urea utilisation by AOA is favored and to estimate the relative importance of ammonium oxidation and urea oxidation in the global ocean.

## 2 Materials and Methods

### 2.1 Sample Collection and Measurements

**FIGURE 1:**
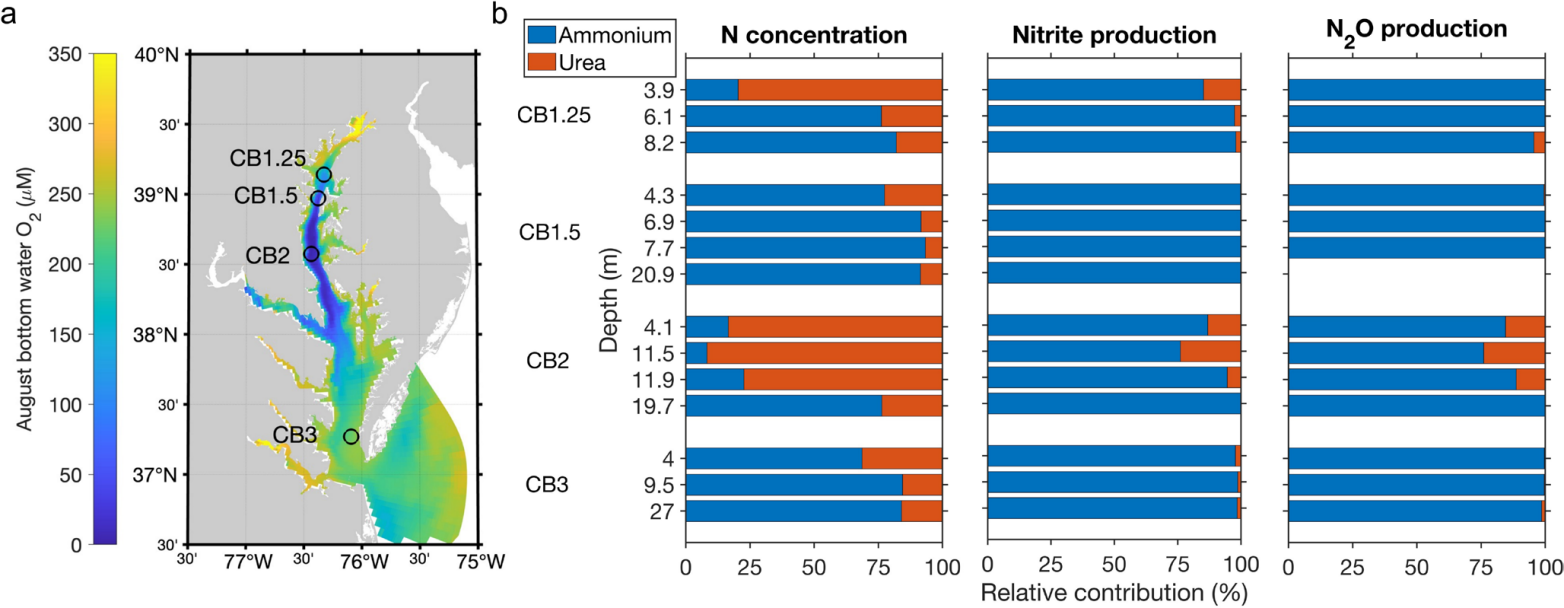
(a) Four sampling stations overlaid on a map of model‐simulated bottom water oxygen in August in Chesapeake Bay (Tang, Da, et al. 2024). Measurements of in situ nitrite and N_2_O production were conducted at all four stations, while substrate manipulation experiments were conducted at stations CB2 and CB1.25. (b) The relative concentration of ammonium versus urea and their contributions to the production of nitrite and N_2_O.

Four stations (CB3, CB2, CB1.5 and CB1.25 shown in Figure 1a) in Chesapeake Bay were sampled August 4–10, 2021, to investigate the contribution of ammonium and urea to nitrite and N_2_O production onboard the R/V Hugh Sharp. Sampling information and biogeochemical properties of these stations can be found in Table S1. These four stations experienced a wide range of environmental conditions from upstream (CB1.25) to downstream (CB3). For example, CB2 and CB1.5 had hypoxic bottom waters, while CB3 and CB1.25 were fully oxygenated (Figure S1). Water samples were collected from a rosette system equipped with twelve 12‐L Niskin bottles and with a CTD profiler (Sea‐Bird Scientific) to record pressure, temperature, salinity, and in situ O_2_ concentration. Triplicate nutrient samples were collected from Niskin bottles into 15 mL Falcon tubes. Concentrations of ammonium were measured shortly after sampling using the fluorometric ortho‐phthalaldehyde method (Holmes et al. 1999). The remaining nutrient samples were kept frozen at −20°C until analysis in the onshore lab. Urea concentrations were determined on frozen samples using the diacetyl monoxime (DAM) method (Chen et al. 2015). The detection limits were 0.1 μM for ammonium and 0.2 μM N for urea. Since urea has two N atoms, urea concentrations were converted to the N concentration of urea for comparison to ammonium throughout the text and figures.

Triplicate N_2_O concentration samples were collected from Niskin bottles into 60 mL serum bottles via Tygon tubing. After overflowing three times the bottle's volume, serum bottles were immediately sealed with butyl stoppers and aluminium crimps. Headspace (3 mL) was created by replacing 3 mL of water with helium gas to allow for the expansion of the liquid during storage. N_2_O samples were then preserved with 100 μL of saturated HgCl_2_ solution. Preserved N_2_O samples were stored in a cold room at a temperature of ~14°C until analysis onshore using previously published protocols (Ji et al. 2015; Tang, Tracey, et al. 2022). Briefly, all N_2_O in the serum bottle was stripped by helium carrier gas into a gas chromatography—isotope ratio mass spectrometer (GC‐IRMS) (Delta V Plus, Thermo) to measure N_2_O concentration and isotope ratio (*m*/*z* = 44, 45, 46). The total amount of N_2_O in the serum bottle was determined by comparing the peak area with N_2_O standards containing a known amount of N_2_O reference gas (0, 0.207, 0.415, 0.623, 0.831, 1.247 nmol N_2_O). The N_2_O concentration was calculated from the amount of N_2_O detected by mass spectrometry divided by the volume of water in each serum bottle. The detection limit and precision of N_2_O concentration measurement were 1.29 and 0.33 nM, respectively.

### 2.2 Measurement of N_2_O and Nitrite Production From Ammonium and Urea

We selected 3–4 depths (surface oxygenated, middle oxycline and bottom low‐oxygen waters) based on the oxygen and nutrient profiles at each station to measure N_2_O and nitrite production under in situ biogeochemical conditions. Water at each depth was collected into 60 mL serum bottles as described above for N_2_O samples. ^15^NH_4_Cl or ^15^N‐urea (Cambridge Isotope Laboratories) was added to reach ~10% of the in situ ammonium or urea concentration. The actual fractions of ^15^N‐labelled ammonium and urea pools ranged from 8.5% to 37.2% and 6.1% to 18.0%, respectively. We performed incubations in a temperature‐controlled dark container to mimic the in situ temperature conditions. Each incubation time course to determine a rate included eight serum bottles with four time points and duplicate bottles at each time point. Duplicate samples were preserved at approximately 0, 4, 8, and 12 h after the tracer addition using 100 μL saturated HgCl_2_ solution. Preserved samples were stored in a dark cold room until analysis in the lab onshore. The N_2_O concentration and nitrogen isotopes from the incubation experiments were measured on a GC‐IRMS as described above for N_2_O concentration samples. N_2_O production rate was calculated by a linear regression based on the progressive increase in mass 45 and 46 N_2_O over the course of the incubation (Trimmer et al. 2016; Bourbonnais et al. 2021). The detection limits were calculated to be 0.0007 and 0.0003 nmol N_2_O L^−1^ d^−1^ for N_2_O production from ammonium and urea, respectively, following the method of Dalsgaard et al. (2012) and Frey et al. (2022).

We measured nitrite production from ammonium (^15^NH_4_^+^) and urea (^15^N‐urea) oxidation using the same samples described above analysed for N_2_O production following the method of Tang, Tracey, et al. (2022). Briefly, after samples were analysed for N_2_O production, 2 mL water was transferred from the serum bottle to a 20‐mL glass vial (Trajan LEAP PAL). After purging with helium for 10 min to remove N_2_O contamination during sample transfer, nitrite in the transferred sample was converted to N_2_O using acetic acid‐treated sodium azide solution (McIlvin and Altabet 2005). The resulting N_2_O concentration and isotope ratio were then measured on the GC‐IRMS. The nitrite production from ammonium or urea oxidation was determined via this equation: Rate=dNO2−15dt×F, where dNO2−15/dt represents the measured NO2−15 concentration change over the course of incubation (dt) (linear regression over all incubation time points), and F represents the calculated fraction of ^15^N (NH4+15NH4+15+NH4+14 or N−urea15N−urea15+N−urea14) in the initial substrate pool (NH_4_^+^ or N‐urea). The detection limits of ammonium oxidation and urea oxidation were 0.09 and 0.02 nmol N L^−1^ day^−1^, respectively. The yield of N_2_O production was estimated by comparing N_2_O production rate with the rate of N_2_O‐producing processes (ammonium oxidation and urea oxidation to nitrite in this study): $\text{yield}\;\left(\%\right)=\frac{\;N_{2}O\;\text{production rate}}{N_{2}O\;\text{production rate}+\text{nitrite production rate}}\times 100$. Urea oxidation: ammonium oxidation ratio was calculated by dividing urea oxidation rate by ammonium oxidation rate (i.e., on the basis of N) and the uncertainty was calculated following the error propagation rule.

### 2.3 Ammonium and Urea Substrate Manipulation Experiments

We evaluated the response of N_2_O and nitrite production to changes in ammonium and urea concentrations and substrate ratio by experimentally adding ammonium or urea into incubation bottles. Waters from the middle depth were more likely to experience fluctuating oxygen and nutrient concentrations, so we selected a middle depth at station CB2 (9.9 m, O_2_ = 179 μM, ammonium = 0.17 μM, urea = 1.12 μM N) and a middle depth at CB1.25 (6.1 m, O_2_ = 197 μM, ammonium = 3.3 μM, urea = 1.03 μM N) for manipulation experiments. The ammonium to urea substrate ratio was manipulated by adding either approximately 2 μM ammonium or 18 μM N‐urea into serum bottles. A substantially higher urea concentration was added to provide enough signal to be detected. ^15^NH_4_^+^ or ^15^N‐urea tracer was then added to serum bottles to obtain ~10% ^15^N of the final ammonium and urea concentration. Concentrations of ammonium and urea substrate and tracers are listed in Table S2. For each incubation time course, incubation in duplicate bottles was stopped at 0, 4, and 8 h after the tracer addition by adding 100 μL saturated HgCl_2_. N_2_O and nitrite production rates were determined via the same approach as the standard rate measurements described above.

### 2.4 Compilation of Ammonium Oxidation and Urea Oxidation Observations in the Global Ocean

Additional observations (Santoro et al. (2017); Arandia‐Gorostidi et al. (2024)) and data from this study were added to a previously compiled dataset of ammonium oxidation and urea oxidation in the global ocean (Wan et al. 2024) to assess the controlling factors on the distribution of ammonium oxidation and urea oxidation. Locations included in the analysis are: the coast of Georgia and South Atlantic Bight (Tolar et al. 2017; Damashek et al. 2018), the Jiulong River Estuary (Tang, Xu, et al. 2022), Chesapeake Bay (Tang, Tracey, et al. 2022), the Gulf of Mexico (Kitzinger et al. 2018), Northeast Pacific (Laperriere et al. 2020; Arandia‐Gorostidi et al. 2024), Equatorial Pacific (Santoro et al. 2017), South China Sea (Wan et al. 2024), North Pacific Subtropical Gyre and Northwestern Pacific (Xu et al. 2019; Wan et al. 2021, 2024), the Antarctic Shelf (Tolar et al. 2017), the Gulf of Alaska (Tolar et al. 2017) and the Arctic Ocean (Shiozaki et al. 2021).

### 2.5 Characterising the Capability of Ammonia Oxidizers to Utilise Urea in Chesapeake Bay and the Global Ocean

The gene *ureC* encodes the alpha subunit of urease, the enzyme that catalyses the hydrolysis of urea. The presence and abundance of the *ureC* gene reflect the capability to utilise urea in organisms and environments. The *amoA* gene (encoding ammonia monooxygenase subunit A) is the functional marker gene of ammonia oxidizers. The ratio of *ureC* to *amoA* has been used to estimate the fraction of ammonia oxidizers containing urease genes (Ahlgren et al. 2017; Santoro et al. 2017; Tolar et al. 2017). We compiled previously published gene abundances of *amoA* and *ureC* in AOA determined by quantitative polymerase chain reaction (qPCR) in the Arctic Ocean, Antarctic Shelf, Gulf of Alaska, and South Atlantic Bight (Alonso‐Saez et al. 2012; Tolar et al. 2017; Shiozaki et al. 2021). In addition, we examined the relative abundance of *ureC* and *amoA* genes in metagenomic samples collected in coastal waters, oligotrophic open ocean, oxygen minimum zones (OMZs), and polar oceans representing a wide range of environmental conditions (Table S3). Metagenomic samples were selected from regions where measurements of either urea and ammonium concentrations or urea oxidation and ammonium oxidation rates were performed concurrently. Collection and analyses of metagenomic samples are detailed below for our newly acquired samples in Chesapeake Bay and North Pacific Subtropical Gyre and can be found for other previously published samples in Table S3 and references therein.

Metagenomic samples from Chesapeake Bay were collected at stations CB3 and CB1.5 in August 2020. Water (1–2 L) was filtered through a 0.22 μm Sterivex filter within 30 min of collection with the CTD rosette. The Sterivex filter was flash‐frozen in liquid nitrogen and then stored in a −80°C freezer until extraction. DNA was extracted using the All‐Prep DNA/RNA Mini Kit (Qiagen) following the manufacturer's protocols. Extracted DNA was quality checked using a bioanalyzer and sequenced on an Illumina Novaseq at the Princeton University Genomics Core Facility.

Metagenomic samples from the North Pacific Subtropical Gyre were collected at stations K8aW and M22W during the KK2007 cruise in 2021 onboard the R/V *Tan Kah Kee*. At each station, seawater was collected from the deep chlorophyll maximum layer (100 and 125 m for station K8aW and M22W, respectively) via Niskin‐X bottles. For each sample, 120 L seawater was pre‐filtered through 200 μm mesh to remove large plankton, and then sequentially filtered through a 142 mm diameter hydrophilic polycarbonate (PC) membrane with a 3 μm pore size and a 142 mm PC membrane with a 0.22 μm pore size (Millipore, GTTP14250). These PC filters were then frozen in liquid nitrogen. Samples from the 0.22–3 μm size fraction were analysed in this study. The environmental DNA was extracted using the FastDNATM Spin Kit (MP Biomedicals) following the manufacturer's protocol. After extraction, the metagenomic libraries were prepared using an Automation System (MGI, MGISP‐960, China) with MGIEasy Universal DNA Library Prep Set (MGI 1000006986, China), using an input of 1 μg of sheared genomic DNA, and then sequenced on the DNBseq platform (Beijing Genomics Institute, Shenzhen, China).

All metagenomic reads (Table S3) were quality filtered with default settings of illumineUtils v. 2.12 (Eren et al. 2013). MEGAHIT v. 1.2.9 (Li et al. 2015) was then used to individually assemble reads with a minimum contig length cutoff of 1000 bp. Eastern Tropical South Pacific samples were co‐assembled due to low sequencing depth. The resulting assemblies were annotated using FunGene for *amoA* and *ureC* with an e‐value cutoff of E‐30 (Eren et al. 2013). Three separate FunGene HMMs were used for *amoA* to capture the differences between the AOA, AOB, and comammox versions of the gene (Fish et al. 2013). Functional gene sequences were extracted using anvi'o v7.1 (Eren et al. 2021) and assigned taxonomy with Kraken v. 2.1.3 as it is especially designed to assign taxonomy to short reads (Wood and Salzberg 2014). All *amoA* and *ureC* genes that could not be classified by Kraken were analysed with a BLASTn query to the nt database (Zhang et al. 2000). Unclassified sequences whose closest hit (using a threshold of 80% nucleotide identity and 50% query coverage) was a known nitrifier were then included in the downstream gene coverage analysis. AOA were the dominant ammonia oxidizers in all the analysed samples. Therefore, only *amoA* and *ureC* hits belonging to AOA were used for downstream analysis.

To avoid mismapping to conserved domains, reads were first mapped to the entire assembly using default settings of bowtie2 (Langmead and Salzberg 2012), and then the average coverage of each gene hit was calculated using anvi'o (Eren et al. 2021). This average coverage is calculated on a per nucleotide basis, so adjustments for gene length were not necessary. Coverage was normalised to the sequencing depth by dividing by the number of million reads in each sample. A ratio of *ureC:amoA* was then calculated for each sample based on this normalised coverage of each gene.

*amoA* and *ureC* genes identified from all metagenomic samples and determined to belong to AOA were aligned with *amoA* and *ureC* reference sequences from cultured marine AOA. *amoA* gene alignments and trees were built using nucleotide sequences. *ureC* genes were first translated from nucleotides to amino acids using Prodigal v. 2.6.3 with default settings (Hyatt et al. 2010), and alignments and trees were based on amino acid sequences (UreC). All alignments were made using mafft v. 7.515 (Katoh and Standley 2013) with a local pairwise alignment and 1000 iterations. Consensus phylogenetic trees for each gene were built using the web‐based IQ‐TREE (Trifinopoulos et al. 2016) with best model selection, 100 bootstraps, and default settings; the best models were GTR+F+I+G4 and LG+I+G4 for the *amoA* and UreC trees, respectively. Trees were visualised using iTOL v6 (Letunic and Bork 2006).

## 3 Results and Discussion

### 3.1 Variable Contribution of Urea to Nitrite and N_2_O Production in Chesapeake Bay

Substrate concentrations (ammonium vs. urea), oxidation rates (ammonium oxidation vs. urea oxidation), and their relative contributions varied across stations and with depth in Chesapeake Bay (Figure 1). Ammonium concentration generally increased with depth at each station, ranging from below the detection limit in surface oxygenated water of CB2 to around 10 μM in the bottom low‐oxygen water of CB1.5 (Figure S1c). The accumulation of ammonium at depth likely resulted from organic matter remineralization in anoxic sediments. Ammonium removal was limited due to low ammonium oxidation rates in the low oxygen bottom water (Figure S2). Urea concentrations were relatively constant across depths, ranging from 0.2 to 1 μM N (Figure S1c). The different vertical distribution of ammonium and urea led to a decreasing fraction of urea in the ammonium+urea pool with increasing depth at each station (Figure 1b). For instance, urea accounted for over 80% of the ammonium+urea pool in surface water but less than 25% of the ammonium+urea pool in bottom water of CB2. In comparison to the high fraction of urea in the ammonium+urea pool (7%–92% with a median of 23%), the contribution of urea to the production of nitrite and N_2_O was substantially smaller, with a median of 1.8% and 0.1%, respectively (Figure 1b). The highest contribution of urea to nitrite and N_2_O production (both at 24%) was observed at 11.5 m of CB2 where the fraction of urea in the ammonium+urea pool was highest (92%). Overall, the contribution of urea to nitrite and N_2_O production increased with the fraction of urea concentration, hinting at a potential substrate control on the relative importance of ammonium versus urea for nitrification and N_2_O production.

### 3.2 Regulation of Nitrite and N_2_O Production in Chesapeake Bay

**FIGURE 2:**
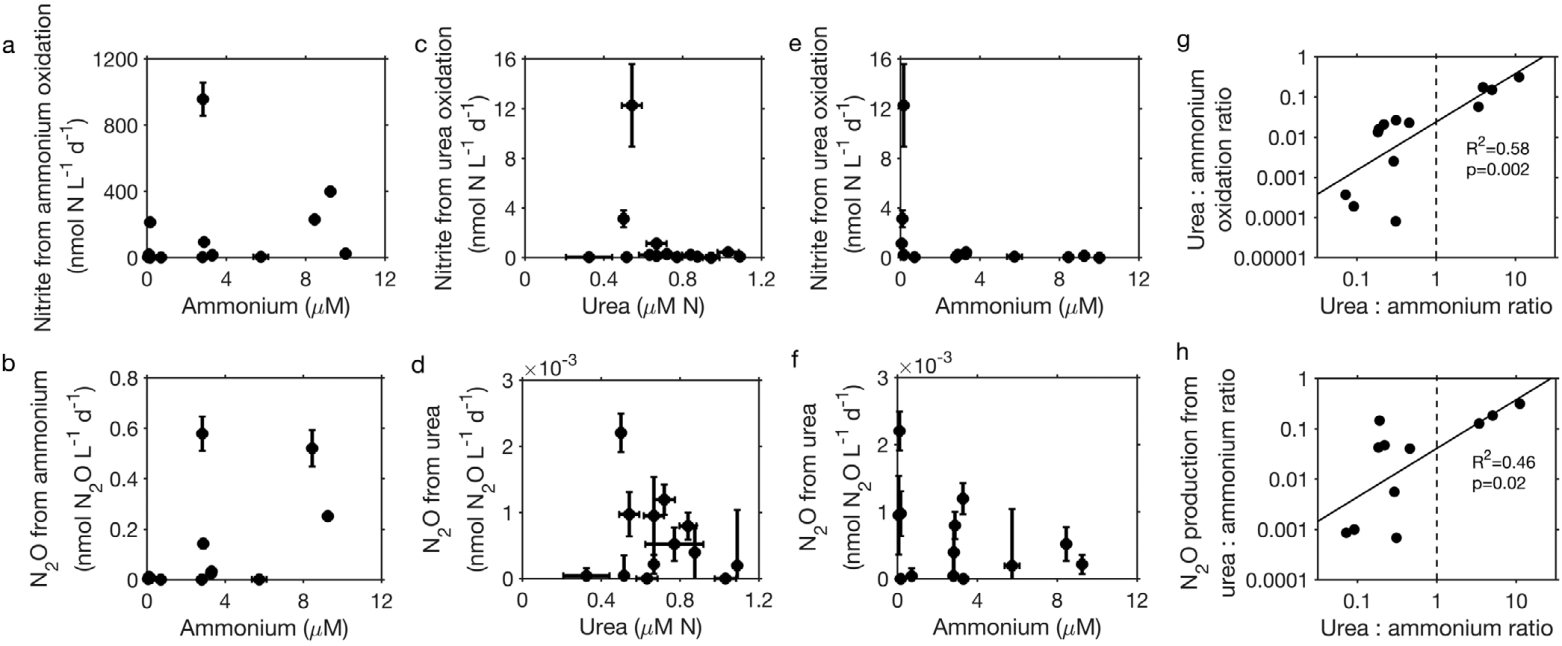
Dependence of in situ production of nitrite and N_2_O from ammonium and urea oxidation on substrate concentrations and substrate ratios in Chesapeake Bay. Vertical error bars represent the uncertainty of linear regression of ^15^NO_2_^−^ or ^15^N‐N_2_O production during the duplicate incubation time courses. Horizontal error bars represent the standard deviations of triplicate measurements of ammonium or urea concentrations.

Ammonium oxidation and associated N_2_O production rates did not show a clear relationship to ammonium concentrations (Figure 2a,b). Similarly, no clear effect of urea concentrations on urea oxidation and associated N_2_O production was found (Figure 2c,d). This lack of apparent substrate regulation on process rates was likely driven by covarying factors such as oxygen. For example, although ammonium accumulated in low oxygen bottom water, ammonium oxidation was limited by low oxygen concentration (e.g., low oxygen bottom water of CB1.5 shown in Figure S1b,c and Figure S2). In addition, urea oxidation decreased under higher ammonium concentrations (Figure 2e). This phenomenon has previously been observed in the ammonium‐enriched Jiulong River Estuary (Tang, Xu, et al. 2022), suggesting a broad inhibitory effect of ammonium on urea utilisation by ammonia oxidizers in estuarine waters. Similarly, urea‐associated N_2_O production generally decreased with higher ammonium concentrations but was more variable (Figure 2f and S3), which may be due to variation in N_2_O production yields. N_2_O production yields from urea oxidation (median of 0.19%) were comparable to N_2_O production yields from ammonium oxidation (median of 0.16%) (slightly higher but not statistically different in Figure S4), likely suggesting similar N_2_O production mechanisms.

Interestingly, the ratio of urea oxidation to ammonium oxidation (*R* ^2^ = 0.58, *p* = 0.002) and the ratio of their associated N_2_O production rates (*R* ^2^ = 0.46, *p* = 0.02) were positively correlated to the ratio of urea to ammonium concentrations (Figure 2g,h), indicating a strong regulation by substrate ratios on the use of urea‐derived N by ammonia oxidizers. Although some samples had higher urea concentrations than ammonium concentrations (urea:ammonium ratio > 1), urea oxidation was always lower than ammonium oxidation (urea oxidation:ammonium oxidation ratio < 1), suggesting a preference of ammonia oxidizers for ammonium over urea in Chesapeake Bay. Lower urea oxidation than ammonium oxidation despite a higher urea concentration has also been found in many other marine environments such as the Northwest Pacific (Xu et al. 2019) and Southern California Bight (Laperriere et al. 2020). This is consistent with the fact that AOA are the dominant ammonia oxidizers in marine systems and demonstrate a preference for ammonium over urea in culture experiments (Qin et al. 2024). This apparent preference for ammonium by marine ammonia oxidizers is an important contrast to β‐AOB, which are commonly the most important ammonia oxidizers in soils and freshwater sediments and show a strong preference for urea in culture experiments (Qin et al. 2024).

**FIGURE 3:**
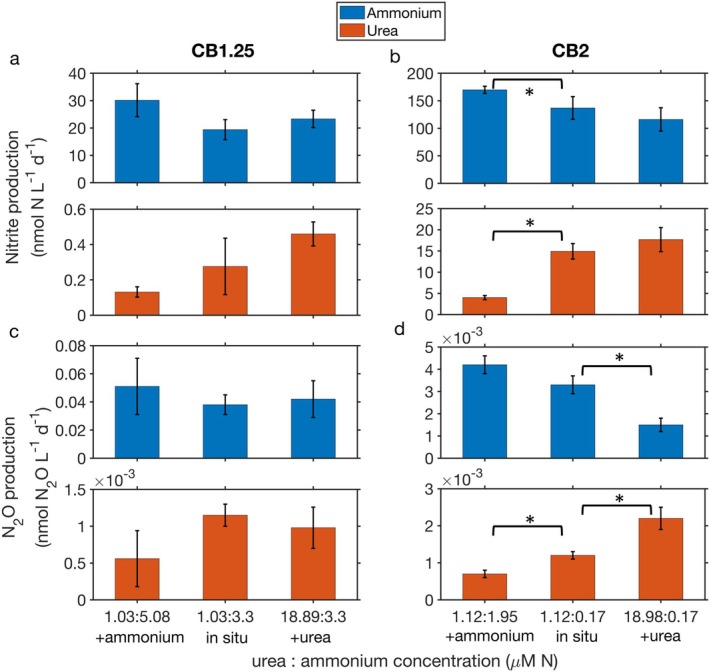
The response of nitrite production (a, b) and N_2_O production (c, d) rates from ammonium (blue bars) and urea (orange bars) to ammonium and urea additions at station CB1.25 (a, c) and CB2 (b, d). In each subplot, middle bars represent incubations conducted under in situ substrate concentrations while first and third bars represent incubations conducted with the addition of ammonium (~2 μM) or urea (~18 μM), respectively. Final substrate ratios (urea:ammonium) noted on the *x* axis. Error bars represent the uncertainty of linear regression of ^15^NO_2_^−^ or ^15^N‐N_2_O production during the duplicate incubation time courses. * indicates significant difference between two treatments at 0.05 level (*t*‐test).

**FIGURE 4:**
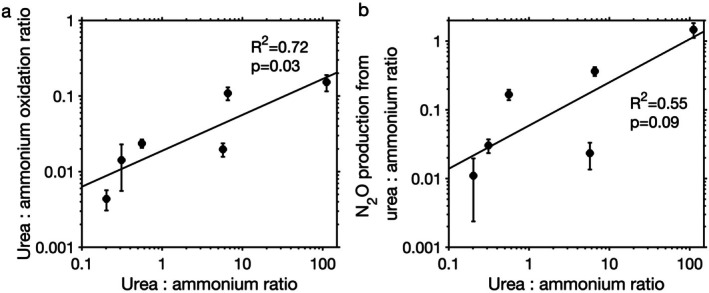
Dependence of the ratio of the production of nitrite (a) and N_2_O (b) from urea and ammonium on the substrate ratio during substrate manipulation experiments in Chesapeake Bay.

To test the impact of substrate ratios on ammonium oxidation and urea oxidation, we measured the production of nitrite and N_2_O from ammonium and urea under manipulated ammonium and urea concentrations while keeping other factors unchanged for samples collected at middle depths of CB2 and CB1.25. Because temperature, salinity, and other covarying environmental factors may also regulate ammonium oxidation and urea oxidation (Figure S5), this approach avoided the potential effect of these covarying factors. Individual substrate addition had variable effects on nitrite and N_2_O production from either ammonium or urea (Figure 3). Results from CB2 showed more significant changes compared to CB1.25, which may be related to the difference in their in situ ammonium concentrations. For example, ammonium addition led to a significant increase in ammonium oxidation rates at CB2 (Figure 3b) with an in situ ammonium concentration of 0.17 μM, which was below the half‐saturation constant of ammonium oxidation observed in Chesapeake Bay (Tang, Fortin, et al. 2024). The increase was not significant at CB1.25 (Figure 3a), where the in situ ammonium concentration was 3.3 μM, which was above the half‐saturation constant of ammonium oxidation. In contrast, ~2 μM ammonium addition inhibited urea oxidation to nitrite with a decrease of 52% and 73% from in situ estimates at CB1.25 and CB2, respectively (Figure 3a,b). Meanwhile, N_2_O production from urea decreased by 52% and 42% from in situ estimates at CB1.25 and CB2, respectively, after ~2 μM ammonium addition (Figure 3c,d). Urea oxidation was not completely inhibited despite the presence of a high ammonium concentration, suggesting direct utilisation of urea by ammonia oxidizers, which has been seen in the Gulf of Mexico and along the Georgia coast (Tolar et al. 2017; Kitzinger et al. 2018). Conversely, a large amount of added urea (~18 μM N‐urea) had a negligible impact on ammonium oxidation and associated N_2_O production except in one case, a significant 54% decrease in N_2_O production at CB2 (Figure 3d). This N_2_O production decrease was possibly driven by isotope dilution of the ammonium tracer by unlabelled ammonium decomposed from the added urea. This isotope dilution effect may have been more obvious at CB2 than CB1.25 because of the lower in situ ammonium concentration at CB2 (0.17 μM at CB2 vs. 3.3 μM at CB1.25). Overall, urea to ammonium substrate ratios showed positive relationships to the ratio of urea oxidation to ammonium oxidation (*R* ^2^ = 0.72, *p* = 0.03) and the ratio of their associated N_2_O production rates (*R* ^2^ = 0.55, *p* = 0.09) (Figure 4). Our substrate manipulation experiments confirmed that the change in urea:ammonium substrate ratios has the potential to affect urea oxidation and ammonium oxidation rates and their relative importance in producing nitrite and N_2_O.

### 3.3 Substrate Effect on Ammonium Oxidation and Urea Oxidation in the Global Ocean

**FIGURE 5:**
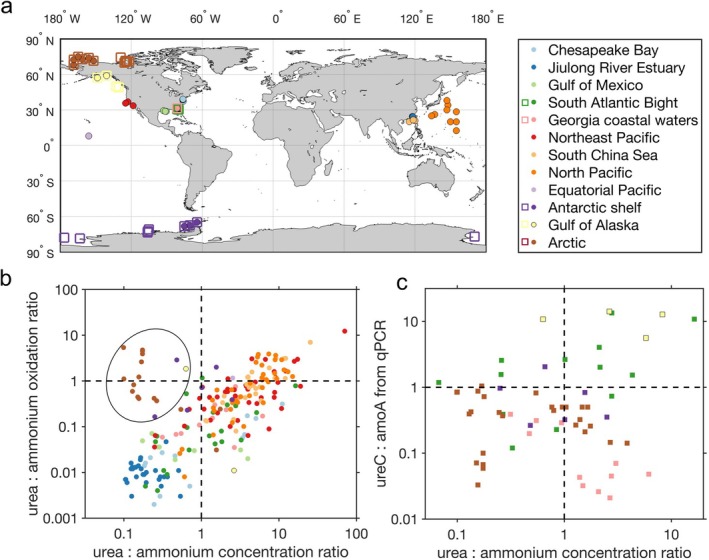
Dependence of urea oxidation:ammonium oxidation rate ratios and the capability of ammonia‐oxidising archaea to utilise urea on urea:ammonium concentration ratios in the global ocean. (a) Map of observed urea oxidation and ammonium oxidation rates (filled circles) and *ureC* and *amoA* gene abundances in AOA from qPCR analyses (open squares) in the global ocean. Observations of nitrification rates and gene abundances overlap in some locations. Observations are consistently colour‐coded by location in (a), (b) and (c). (b) Relationship between urea oxidation:ammonium oxidation rate ratios and urea:ammonium concentration ratios in different ocean regions. The black oval highlights measurements with relatively larger urea oxidation:ammonium oxidation ratios despite their low urea:ammonium concentration ratios (identified outside the two standard deviations of the robust linear regression fit to the dataset), which were mostly observed in the polar oceans. (c) *ureC*:*amoA* gene abundance ratios in AOA determined by qPCR compiled from previous studies (Alonso‐Saez et al. 2012; Tolar et al. 2017; Shiozaki et al. 2021).

To assess the ubiquity of substrate control on ammonium oxidation and urea oxidation that we observed in Chesapeake Bay, we compiled measurements of ammonium oxidation and urea oxidation rates in the global ocean (Figure 5a and Supplementary Dataset in Data Availability). Ammonium oxidation rates generally increase with ammonium concentrations (*r* = 0.65, *p* < 0.01) while urea oxidation rates show a weaker positive relationship with urea concentrations at the global scale (*r* = 0.34, *p* < 0.01) (Figure S6). This is partly because high urea concentrations are often associated with high ammonium concentrations (Figure S7), which tend to inhibit urea oxidation as seen in Chesapeake Bay (Figure 2) and in Jiulong River Estuary (Tang, Xu, et al. 2022). The ratio of urea to ammonium concentrations is a good predictor of the relative importance of urea and ammonium to nitrite production (Figure 5b). The ratio of urea oxidation to ammonium oxidation generally increases with the ratio of urea to ammonium concentrations across different marine environments from eutrophic estuaries to oligotrophic open oceans ($\log_{10}y=1.113\times \log_{10}x-0.97$, *R* ^2^ = 0.66, *p* < 0.01), with some anomalous observations in the polar oceans (Figure S8), as previously suggested (Wan et al. 2024). This positive relationship holds in open oceans alone after removing data collected from estuaries and polar regions ($\log_{10}y=1.064\times \log_{10}x-0.893$, *R* ^2^ = 0.6, *p* < 0.01).

Approximately 78% of the observations showed higher ammonium oxidation rates compared to urea oxidation (Figure 5b), confirming the preference of most marine ammonia oxidizers for ammonium. However, higher urea oxidation rates compared to ammonium oxidation have been observed in the Southern California Bight, South China Sea, and North Pacific subtropical gyre, which could be partially explained by a relatively higher urea availability (urea:ammonium ratio > ~4). The high contribution of urea to nitrite production is also more frequently found in the deep ocean where urea concentrations are often higher than ammonium concentrations, despite both concentrations being quite low (Figure S9). Polar oceans are an exception to this relationship. Despite the low urea to ammonium concentration ratio (< 1), urea oxidation unexpectedly outpaced ammonium oxidation in the Antarctic shelf (Tolar et al. 2017) and in the Arctic Ocean (Shiozaki et al. 2021) (data points within the black circle in Figure 5b). These outliers suggest that ammonia oxidizers in polar oceans may be more capable of utilising urea or even prefer urea over ammonium.

### 3.4 Capability of Ammonia Oxidizers to Utilise Urea Across Substrate Gradients in the Global Ocean

To evaluate the capability of ammonia oxidizers to utilise urea, we analysed the previously reported gene abundances of *ureC* and *amoA* (functional genes for urea hydrolysis and ammonium oxidation, respectively) determined with qPCR across a wide range of marine environments. The *ureC*:*amoA* ratio in Nitrososphaerota (phylum of the Archaea containing marine AOA, formerly known as Thaumarchaeota) was found to be higher in Arctic water (median: 0.28) and Antarctic water (median: 0.35) than in Georgia coastal water (median: 0.16) (Alonso‐Saez et al. 2012; Tolar et al. 2017; Shiozaki et al. 2021) (Figures 5c and S10). This may help to explain the high urea oxidation:ammonium oxidation ratios despite low urea:ammonium ratios observed in polar oceans (Figure 5b). However, abnormally high *ureC*:*amoA* ratios in Nitrososphaerota were found in the South Atlantic Bight (median: 1.79) and Gulf of Alaska (median: 8.22), and did not always result in higher ratios of urea oxidation to ammonium oxidation. These abnormally high *ureC*:*amoA* ratios in the South Atlantic Bight and Gulf of Alaska may be caused by primer biases, for example, non‐specific amplification when targeting Nitrososphaerota *ureC* genes (Tolar et al. 2017). Although *ureC* gene abundance was quantified using the same qPCR primers developed by Alonso‐Saez et al. (2012) across all studies, different primer sets were used for quantifying *amoA* gene abundance. Alonso‐Saez et al. (2012) and Tolar et al. (2017) used primers designed by Wuchter et al. (2006) while Shiozaki et al. (2021) used primers designed by Francis et al. (2005) and Beman et al. (2008). This primer difference could also have induced uncertainties in comparing *ureC*:*amoA* ratios across studies. Overall, there was no significant relationship between qPCR‐derived *ureC*:*amoA* ratios and urea:ammonium ratios (Figure 5c).

To avoid the issue of primer bias in qPCR, we examined the abundance of *amoA* and *ureC* genes in metagenomic samples collected from representative ocean regions (estuarine and coastal waters, subtropical gyres, OMZs, and the Southern Ocean) (Table S3). Although metagenomic samples have been collected in many parts of the ocean (Chen et al. 2024), few of them contain urea and ammonium concentration data. One of the main goals of this study is to assess how ammonia oxidizers' ability to utilise urea varies across urea and ammonium substrate gradients rather than in all the metagenomic samples globally (which itself is interesting and deserves future investigation). Therefore, we focused on analysing metagenomic samples that were collected along with measurements of urea and ammonium concentrations or urea and ammonium oxidation rates (Table S3).

**FIGURE 6:**
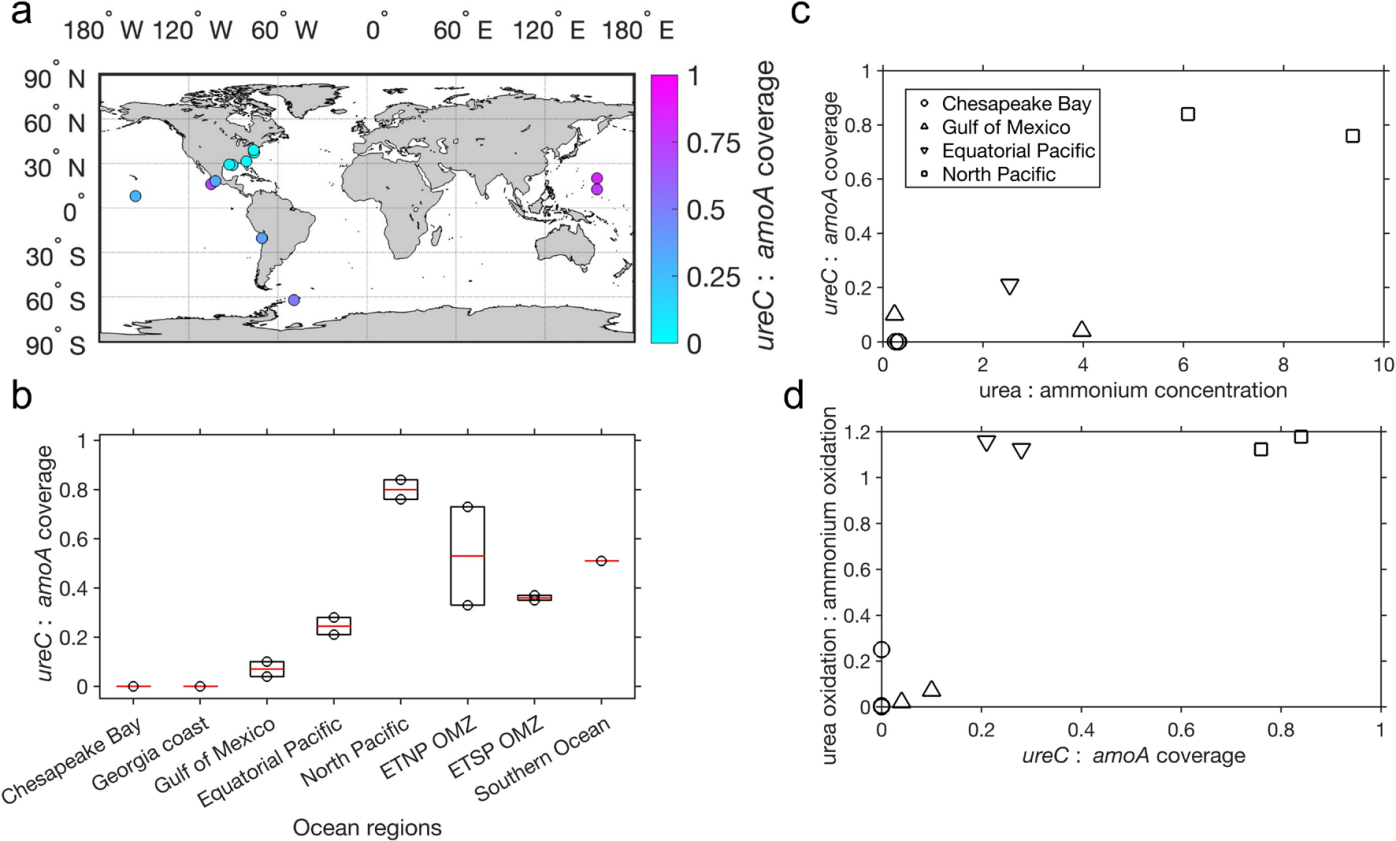
(a) The distribution of *ureC*:*amoA* gene coverage ratios in metagenomic samples. (b) Boxplot of *ureC*:*amoA* ratios in AOA across different ocean regions. (c) The relationship between urea:ammonium concentration ratios and *ureC*:*amoA* gene coverage ratios (*y* = 0.083 × *x* − 0.018, *R* ^2^ = 0.89, *p* < 0.01). (d) The relationship between *ureC*:*amoA* gene coverage ratios and urea oxidation:ammonium oxidation rate ratios (*y* = 1.449 × *x* − 0.014, *R* ^2^ = 0.89, *p* < 0.01). Normalised coverages of *amoA* and *ureC* genes are shown in the figure. Data presented in (c) and (d) are obtained from concurrent collections or measurements of substrate concentrations, metagenomic samples and nitrification rates at the same locations. Some data points from Chesapeake Bay are overlapping.

Since AOA were the dominant ammonia oxidizers in all the analysed global metagenomic samples, we focused on evaluating the abundance of *amoA* and *ureC* genes in AOA using gene coverage per million reads (see coverage analysis in Methods). Diverse *amoA* and *ureC* genes of AOA were found in the global metagenomic samples (Figures S11 and S12). AOA *amoA* and *ureC* coverages varied spatially (Figure S13), leading to substantial variations in the ratios of *ureC* to *amoA* in AOA (Figure 6a,b). For example, the median coverages of AOA *amoA* in Chesapeake Bay and Georgia coastal waters were 0.55 and 2.44, respectively, while no AOA *ureC* genes were identified from these metagenomic assemblies, resulting in *ureC:amoA* ratios of zero. Consistent with the low coverage of *ureC* in metagenomic assemblies, metagenome‐assembled genomes (MAGs) of AOA in our Chesapeake Bay samples did not contain *ureC* genes. Thus, it is likely most of the urea oxidation was conducted by AOB in Chesapeake Bay. *amoA* and *ureC* associated with AOB were indeed found in Chesapeake Bay although at low abundance (data not shown). However, we can't exclude the possibility that AOA oxidised urea‐derived N that was produced from urea decomposition by other microbes (high *ureC* coverage of the whole microbial community shown in Table S4). A previous study found no *ureC* genes in AOA MAGs binned in the Jiulong River Estuary (Zou et al. 2020). In addition, *ureC:amoA* ratios of AOA in metatranscriptomic samples collected from Georgia coastal waters were also low, with a maximum of 0.04 (Tolar et al. 2017). Therefore, low frequencies of *ureC* genes in AOA were generally found in estuarine and coastal waters where ratios of urea:ammonium concentrations and urea oxidation:ammonium oxidation rates were also low (lower left panel of Figure 5b). Nevertheless, decomposition of urea by other organisms could potentially supply ammonium to AOA, as implied by high *ureC* coverages of the whole microbial community (Table S4) and suggested by previous studies (Koch et al. 2015; Pachiadaki et al. 2017; Arandia‐Gorostidi et al. 2024).

*ureC*:*amoA* ratios increased from coastal oceans toward open oceans and OMZs (Figure 6b). This was accompanied by a shift in AOA genotypes along the inshore‐offshore gradient: *Nitrosopumilus*‐like AOA dominating in estuaries and *Ca*. Nitrosopelagicus‐like AOA becoming abundant in open ocean surface waters, as has been observed in Qin et al. (2020). In the Gulf of Mexico, the median *ureC*:*amoA* ratio was 0.07, similar to the previous estimate that 10%–15% of Nitrososphaerota contain a urease (Kitzinger et al. 2018). The median *ureC*:*amoA* ratio was 0.25 in the equatorial Pacific, within the lower range of the previous estimate of 0.22–0.55 (Santoro et al. 2017). This difference may be affected by the methods used to identify *amoA* and *ureC* genes (e.g., the use of FunGene HMMs to identify *ureC* and a higher nucleotide identity cutoff for blastn in our samples). The *ureC*:*amoA* ratio in the North Pacific Subtropical Gyre reached a median of 0.8 where high ratios of urea:ammonium concentrations and urea:ammonium oxidation rates were observed (upper right portion of Figure 5c). This high *ureC*:*amoA* ratio is consistent with a previous study, showing that a large portion (60%–100%) of Nitrososphaerota contains *ureC* genes in the Southern California Bight (Ahlgren et al. 2017). Although urea oxidation has not been measured in the OMZs, median *ureC*:*amoA* ratios were found to be 0.53 and 0.33 in the Eastern Tropical North Pacific (ETNP) and Eastern Tropical South Pacific (ETSP) OMZs, respectively, suggesting potentially a large contribution of urea to support AOA growth, and the production of nitrite and N_2_O in these hotspots of nitrogen loss and N_2_O emissions (DeVries et al. 2013; Yang et al. 2020).

Moderate *ureC*:*amoA* ratios existed in the Southern Ocean (0.51). Simultaneous collection/measurements of metagenomic samples and concentrations of urea and ammonium are lacking in the Arctic Ocean. The limited observations prevented us from examining the exact mechanisms driving the elevated urea oxidation:ammonium oxidation ratios in polar oceans despite the low urea:ammonium concentration ratios (data shown in the black oval in Figure 5b). We hypothesize that the elevated *ureC*:*amoA* ratio of AOA in polar oceans may be one of the drivers, but this remains to be explored.

There was a positive relationship between *ureC*:*amoA* ratios and urea:ammonium concentration ratios across the ocean geography from estuaries to open oceans (Figure 6c). In comparison, urea or ammonium concentration alone was not a good predictor of *ureC*:*amoA* ratios (Figure S14). In addition, urea oxidation:ammonium oxidation ratios positively correlated with *ureC*:*amoA* ratios (Figure 6d). Therefore, substrate ratios may regulate the community composition of ammonia oxidizers, their ability to utilise urea, and subsequently the contribution of urea to nitrite and N_2_O production. However, more simultaneous measurements of substrate concentrations, nitrification rates, and molecular analyses of *ureC* and *amoA* gene abundance are required to validate this pattern in the broad ocean regions. In addition, we focus on the analysis of AOA because of their dominance in all the analysed global metagenomic samples. AOB can be abundant in estuarine waters, and their capability to utilise urea may also be affected by the urea:ammonium concentration ratios, which remains to be determined. Finally, the effect of other environmental factors (e.g., temperature) on the observed correlations deserves further investigation.

## 4 Conclusions and Implications

Nitrite oxidation is frequently reported to be higher than ammonium oxidation (Tang et al. 2023), indicating a missing source of nitrite. Combining new observations from Chesapeake Bay with previous measurements in the global ocean, we revealed that urea is an important substrate for nitrification and nitrite production, despite ammonium being the preferred substrate in most marine environments. The absolute nitrite production rate from urea correlates with urea concentration, while the relative importance of urea to nitrification varies spatially with the ratio of urea to ammonium concentrations (lower in estuarine and coastal waters while higher in open oceans), which may contribute to variability in the relationship between ammonium concentration and ammonium oxidation rates (Tang et al. 2023). Although the number of observations of N_2_O production from urea is limited, we hypothesize that the relative contribution of urea to N_2_O production may be similar to the relative contribution of urea to nitrite production because of our measured comparable N_2_O production yields from urea and ammonium. We encourage future observations of N_2_O production from urea to better constrain the total N_2_O production and emissions. In addition, the genetic capability to utilize urea (i.e., *ureC*:*amoA* ratio) has been shown to vary among ammonia oxidizers and across marine environments (Ahlgren et al. 2017; Kitzinger et al. 2018; Qin et al. 2024), although the mechanisms were unclear. Here, we suggest that the ratio of urea to ammonium concentrations helps to explain this variation and is a key factor in shaping different assemblages of AOA to utilise urea, which has implications for assessing the evolution of diverse AOA to utilise different substrates.

Substrate concentration is a fundamental constraint on the absolute rate of nitrification. Our study illustrates the predictive potential of substrate ratios to estimate the relative contributions of urea and ammonium to nitrite and N_2_O production, which has not previously been recognised. Future additional measurements of urea and ammonium concentrations can largely expand our understanding of the distribution of urea oxidation and ammonium oxidation and their contribution to N_2_O production. This correlation framework could inspire future studies to examine the mechanisms and develop quantitative models, which could be further used to evaluate changes in nitrification under human perturbations and future climate. For instance, urea concentrations in estuaries could increase due to the rise in urea fertiliser usage (Glibert et al. 2006), possibly selecting for an ammonia‐oxidising community that uses or even prefers urea. These changing factors would modulate the concentration of urea and ammonium, the abundance and activities of nitrifiers and their use of urea, eventually affecting the forms of bioavailable nitrogen, marine productivity, and N_2_O production in the ocean.

## Author Contributions

**Weiyi Tang:** conceptualization, data curation, formal analysis, investigation, visualisation, writing – original draft. **Catherine Hexter:** formal analysis, writing – review and editing. **Rongbo Dai:** formal analysis, writing – review and editing. **Samantha G. Fortin:** formal analysis, writing – review and editing. **John C. Tracey:** investigation, writing – review and editing. **Naomi Intrator:** investigation, writing – review and editing. **Moriah A. Kunes:** investigation, writing – review and editing. **Xianhui S. Wan:** investigation, writing – review and editing. **Amal Jayakumar:** investigation, writing – review and editing. **Dalin Shi:** investigation, writing – review and editing. **Bess B. Ward:** funding acquisition, supervision, writing – review and editing.

## Conflicts of Interest

The authors declare no conflicts of interest.

## Supporting information


**Data S1:** emi70187‐sup‐0001‐Supinfo.pdf.

## Data Availability

Data presented in this study have been deposited into Zenodo repository: https://doi.org/10.5281/zenodo.15223777. Accession numbers for newly generated and previously published metagenomic datasets used in this study are listed in Table S3.
