# Supplementary material for: Substrate Effect on the Contribution of Ammonium and Urea to Marine Nitrification and Nitrous Oxide Production
Source: Environ Microbiol. 2025 Oct 6;27(10):e70187. doi: 10.1111/1462-2920.70187 (PMC12500505; doi:10.1111/1462-2920.70187)
Supplement: Supplementary file 1 — Data S1: emi70187‐sup‐0001‐Supinfo.pdf. [file EMI-27-e70187-s001.pdf]

Supporting Information

Substrate effect on the contribution of ammonium and urea to marine nitrification and nitrous oxide production

Weiyi Tang[1, 2, *], Catherine Hexter[1], Rongbo Dai[3], Samantha G. Fortin[1], John C. Tracey[1, 4], Naomi Intrator[1], Moriah A. Kunes[1], Xianhui Sean Wan[1, 3], Amal Jayakumar[1], Dalin Shi[3], Bess B. Ward[1]

[1]Department of Geosciences, Princeton University, Princeton, NJ, United States
[2]College of Marine Science, University of South Florida, St Petersburg, FL, United States
[3]State Key Laboratory of Marine Environmental Science, College of Ocean and Earth Sciences, Xiamen University, Xiamen, China
[4]Currently at US Department of Energy, Fusion Energy Sciences, Germantown, MD, United States

[*]Correspondence to: weiyitang@usf.edu

Table S1. Biogeochemical properties of the sampling locations.

Station	Date	Latitude	Longitude	Depth (m)	Temperature (℃)	Salinity	O_2 (μM)	Ammonium (μM)	Urea (μM N)	N_2O (nM)	Nitrite (μM)
CB3	8/4/21	37.2701	-76.0924	3.98	25.14	23.57	202	0.71	0.32	8.97	0.12
CB3	8/4/21	37.2701	-76.0924	9.50	24.42	26.39	169	2.80	0.52	9.10	0.20
CB3	8/4/21	37.2701	-76.0924	27.00	23.09	28.31	144	5.74	1.09	9.94	0.38
CB2	8/5/21	38.5733	-76.4370	4.11	25.31	9.34	226	0.1	0.50	9.54	0.18
CB2	8/5/21	38.5733	-76.4370	11.50	26.58	15.98	117	0.06	0.67	10.23	0.04
CB2	8/5/21	38.5733	-76.4370	11.91	26.57	18.36	45	0.16	0.54	10.48	0.47
CB2	8/5/21	38.5733	-76.4370	19.70	26.34	21.03	4	2.83	0.88	5.26	2.53
CB1.5	8/8/21	38.9709	-76.3768	4.30	25.47	9.08	187	2.88	0.84	7.45	0.48
CB1.5	8/8/21	38.9709	-76.3768	6.87	25.86	12.50	66	8.45	0.77	4.88	0.81
CB1.5	8/8/21	38.9709	-76.3768	7.74	25.84	14.39	23	9.25	0.67	3.68	0.54
CB1.5	8/8/21	38.9709	-76.3768	20.87	26.31	19.85	2	10.01	0.94	1.08	0.09
CB1.25	8/10/21	39.1398	-76.3273	3.92	27.32	6.53	308	0.16	0.63	9.26	0.30
CB1.25	8/10/21	39.1398	-76.3273	6.14	26.44	7.43	197	3.23	1.03	8.84	0.43
CB1.25	8/10/21	39.1398	-76.3273	8.24	26.53	8.29	188	3.28	0.72	8.79	0.30

Table S2. Concentrations of ammonium (NH_4^+) and urea in substrate manipulation experiments. Unit: μM N.

Station	Depth (m)	Tracer	Treatment	in situ NH_4^+	added NH_4^+	$^{15}NH_4^+$ tracer	Total NH_4^+	in situ urea	added urea	^{15}N-urea tracer	Total urea	urea : NH_4^+
CB1.25	6.1	$^{15}NH_4^+$	add NH_4^+	3.3	1.78	0.71	5.79	1.03	0	0	1.03	1.03 : 5.79
			control	3.3	0	0.36	3.66	1.03	0	0	1.03	1.03 : 3.66
			add urea	3.3	0	0.36	3.66	1.03	17.86	0	18.89	18.89 : 3.66
		^{15}N-urea	add NH_4^+	3.3	1.78	0	5.08	1.03	0	0.07	1.10	1.1 : 5.08
			control	3.3	0	0	3.3	1.03	0	0.07	1.10	1.1 : 3.3
			add urea	3.3	0	0	3.3	1.03	17.86	1.78	20.67	20.67 : 3.3
CB2	9.9	$^{15}NH_4^+$	add NH_4^+	0.17	1.78	0.18	2.13	1.12	0	0	1.12	1.12 : 2.13
			control	0.17	0	0.03	0.2	1.12	0	0	1.12	1.12 : 0.2
			add urea	0.17	0	0.03	0.2	1.12	17.86	0	18.98	18.98 : 0.2
		^{15}N-urea	add NH_4^+	0.17	1.78	0	1.95	1.12	0	0.07	1.19	1.19 : 1.95
			control	0.17	0	0	0.17	1.12	0	0.07	1.19	1.19 : 0.17
			add urea	0.17	0	0	0.17	1.12	17.86	1.78	20.76	20.76 : 0.17

Table S3. Metagenomic sample information for *amoA* and *ureC* analysis. ETNP OMZ: Eastern Tropical North Pacific Oxygen Minimum Zone; ETSP OMZ: Eastern Tropical South Pacific Oxygen Minimum Zone.

Region	Station	Date	Latitude	Longitude	Depth (m)	Size fraction (µm)	Number of reads	Accession number	Reference
Chesapeake Bay	CB3	8/27/20	37.27	-76.09	4	>0.22	233708066	PRJNA1151642	this study
Chesapeake Bay	CB3	8/27/20	37.27	-76.09	24	>0.22	273623479	PRJNA1151642	this study
Chesapeake Bay	CB1.5	8/29/20	38.97	-76.38	4.3	>0.22	237003416	PRJNA1151642	this study
Chesapeake Bay	CB1.5	8/29/20	38.97	-76.38	12.4	>0.22	259986367	PRJNA1151642	this study
Chesapeake Bay	CB1.5	8/29/20	38.97	-76.38	23	>0.22	215344876	PRJNA1151642	this study
Georgia coast during day	Marsh Landing	August 2009	31.42	-81.28	0.2	0.22-3	6258053	SAMN12212021	Damashek et al. (2019)
Georgia coast during night	Marsh Landing	August 2009	31.42	-81.28	0.2	0.22-3	6324184	SAMN12212029	Damashek et al. (2019)
Gulf of Mexico	Station 1	7/24/16	28.87	-90.48	18	>0.22	72032256	SAMN10227781	Kitzinger et al. (2018)
Gulf of Mexico	Station 2	7/28/16	29.30	-92.80	14	>0.22	58352508	SAMN10227779	Kitzinger et al. (2018)
Equatorial Pacific	Station 3	10/8/11	8.00	-156.00	150	>0.2	44632019	SAMN05422039	Santoro et al. (2017)
Equatorial Pacific	Station 3	10/8/11	8.00	-156.00	300	>0.2	43881883	SAMN05421567	Santoro et al. (2017)
North Pacific	M22W	1/11/21	20.00	155.00	125	0.22-3	200052006	SRR32817908	this study
North Pacific	K8aW	1/17/21	12.50	155.00	100	0.22-3	200003762	SRR32817907	this study
ETNP OMZ	PS2	3/27/18	16.00	-105.00	95	>0.22	86903174	SAMN36908805	Fortin et al. (2024)
ETNP OMZ	PS3	3/8/18	18.00	-102.00	800	>0.22	69757396	SAMN36908813	Fortin et al. (2024)
ETSP OMZ	BB2	7/17/13	-20.46	-70.68	80	>0.22	11408338	mgm4842563.3	Sun et al. (2019)
ETSP OMZ	BB2	7/16/13	-20.52	-70.71	200	>0.22	10121052	mgm4842564.3	Sun et al. (2019)
Southern Ocean	Tara 85	1/7/11	-62.22	-49.21	790	0.22-3	165789358	SAMEA2621551	Salazar et al. (2019)

Table S4. *amoA* and *ureC* coverages of metagenomic samples shown in Table S3. Unit of coverage: gene occurrence per million reads. * mostly from phytoplankton and heterotrophic bacteria.

Region	Station	Depth (m)	AOA *amoA*	AOA *ureC*	AOA *ureC*:*amoA*	Whole community *ureC**
Chesapeake Bay	CB3	4	0.10	0.00	0.00	9.43
Chesapeake Bay	CB3	24	0.08	0.00	0.00	15.00
Chesapeake Bay	CB1.5	4.3	0.55	0.00	0.00	7.05
Chesapeake Bay	CB1.5	12.4	3.84	0.00	0.00	13.69
Chesapeake Bay	CB1.5	23	4.33	0.00	0.00	16.15
Georgia coast	Marsh Landing	0.2	2.16	0.00	0.00	19.81
Georgia coast	Marsh Landing	0.2	2.71	0.00	0.00	22.30
Gulf of Mexico	Station 1	18	9.93	1.02	0.10	17.05
Gulf of Mexico	Station 2	14	6.06	0.25	0.04	14.09
Equatorial Pacific	Station 3	150	6.80	1.45	0.21	35.75
Equatorial Pacific	Station 3	300	10.07	2.86	0.28	47.03
North Pacific	M22	125	8.29	6.99	0.84	51.15
North Pacific	K8a	100	3.13	2.37	0.76	52.70
ETNP OMZ	PS2	95	1.75	1.28	0.73	5.05
ETNP OMZ	PS3	800	1.42	0.46	0.33	14.33
ETSP OMZ	BB2	80	3.66	1.30	0.35	16.54
ETSP OMZ	BB2	200	0.17	0.06	0.37	34.14
Southern Ocean	Tara 85	790	4.97	2.55	0.51	23.16

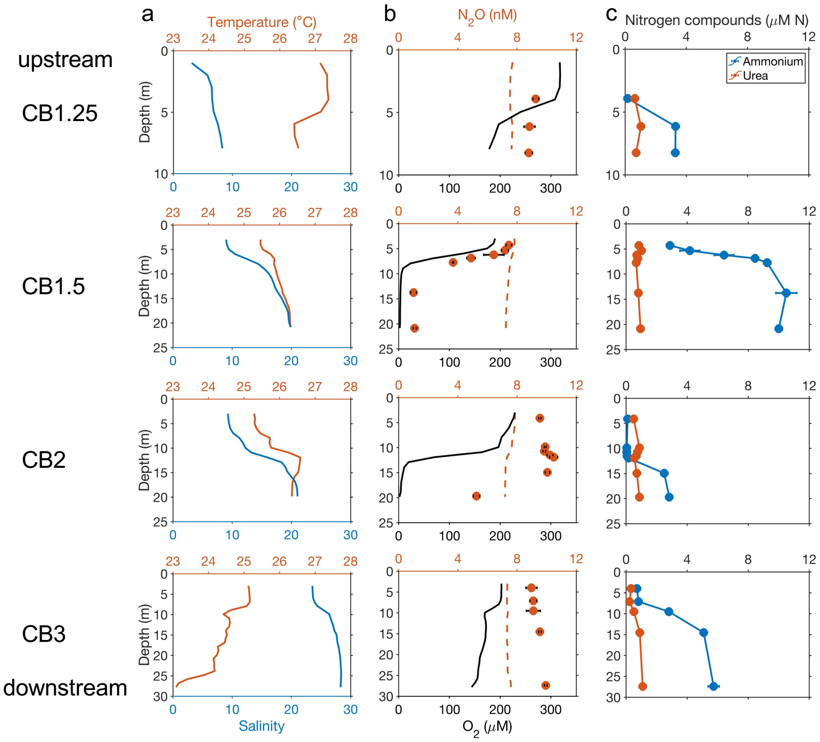

Figure S1. Depth profiles of temperature and salinity (a), oxygen and N_2O (b), ammonium and urea concentrations (c) observed during the 2021 August cruise. The equilibrium N_2O concentration in (b) is shown as orange dashed lines. Error bars represent standard deviation of replicate samples.

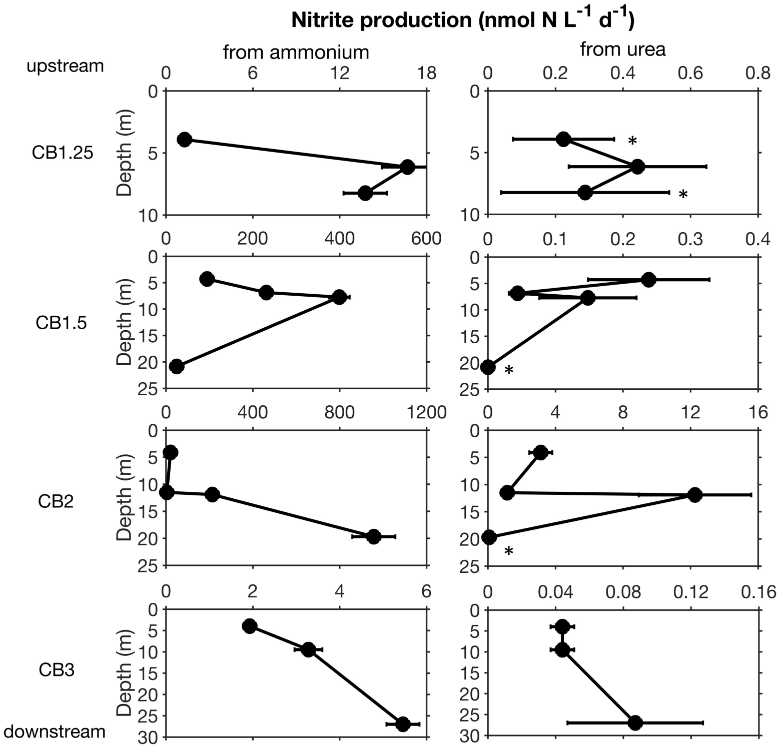

Figure S2. Depth profiles of nitrite production rates from ammonium and urea (i.e., ammonium and urea oxidation) at four stations. Horizontal error bars represent the uncertainty of linear regression of $^{15}NO_2^-$ production during the duplicate incubation time courses. Rates not significantly different from 0 are denoted by *.

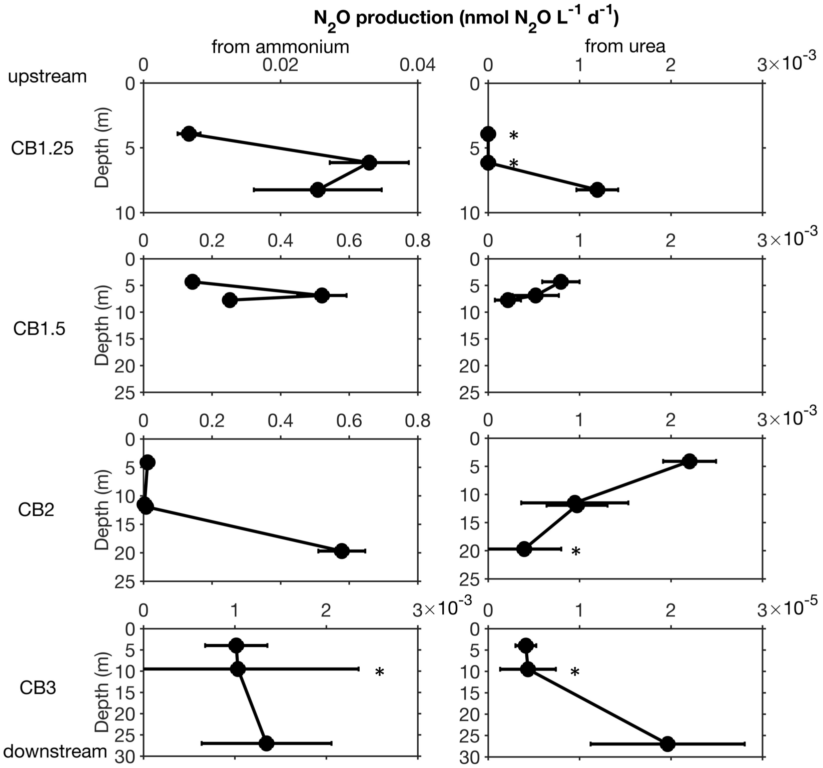

Figure S3. N_2O production rates from ammonium and urea during the oxidation to nitrite. N_2O production was not measurable at bottom water of CB1.5 because N_2O concentrations were the below detection limit, which was likely caused by larger N_2O reduction than production in the low oxygen water (Tang et al., 2022). Horizontal error bars represent the uncertainty of linear regression of ^{15}N-N_2O production during the duplicate incubation time courses. Rates not significantly different from 0 are denoted by *.

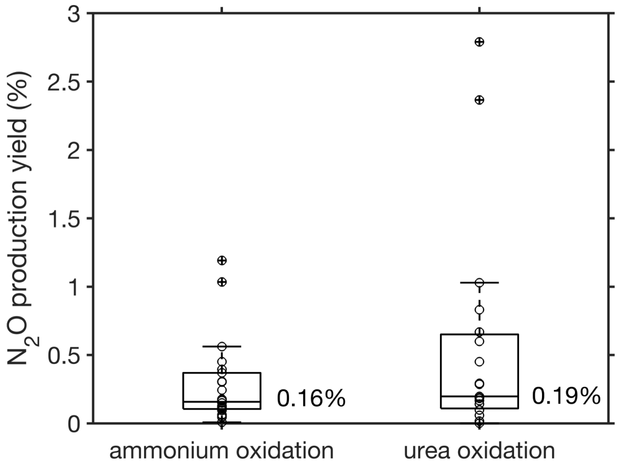

Figure S4. Comparison of N_2O production yields from ammonium oxidation and urea oxidation. Median values are shown for the boxplots. The bottom and top of each box are the 25th and 75th percentiles of the observations, respectively. The error bars represent 1.5 times the interquartile range away from the bottom or top of the box, with + signs showing outliers beyond that range. Individual data points are shown as circles.

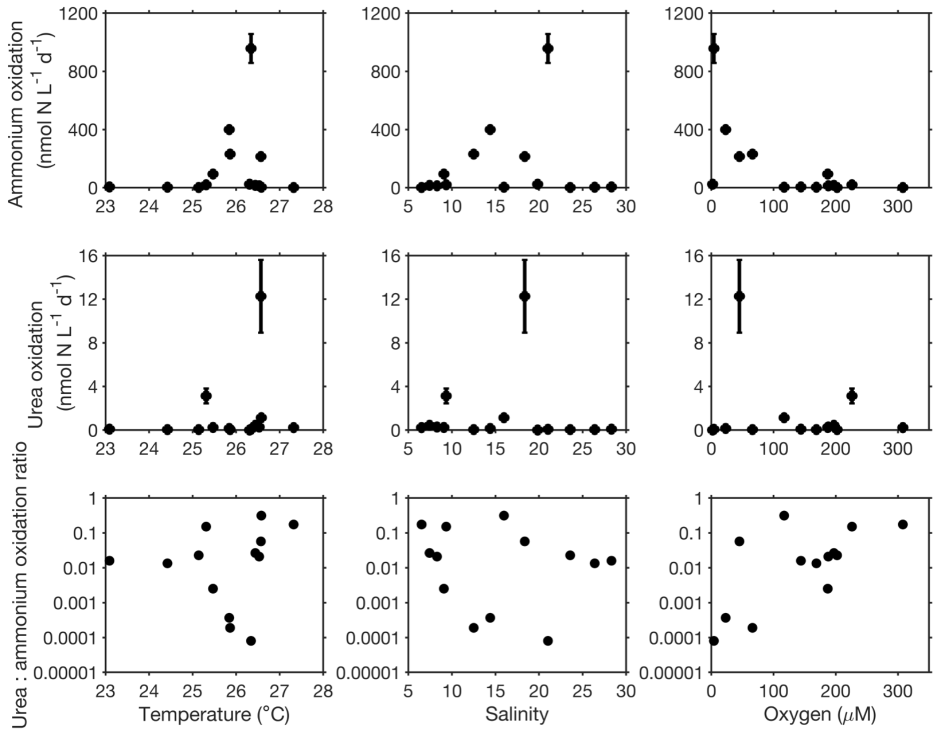

Figure S5. The relationship between *in situ* environmental conditions (temperature, salinity and oxygen) and nitrification rates.

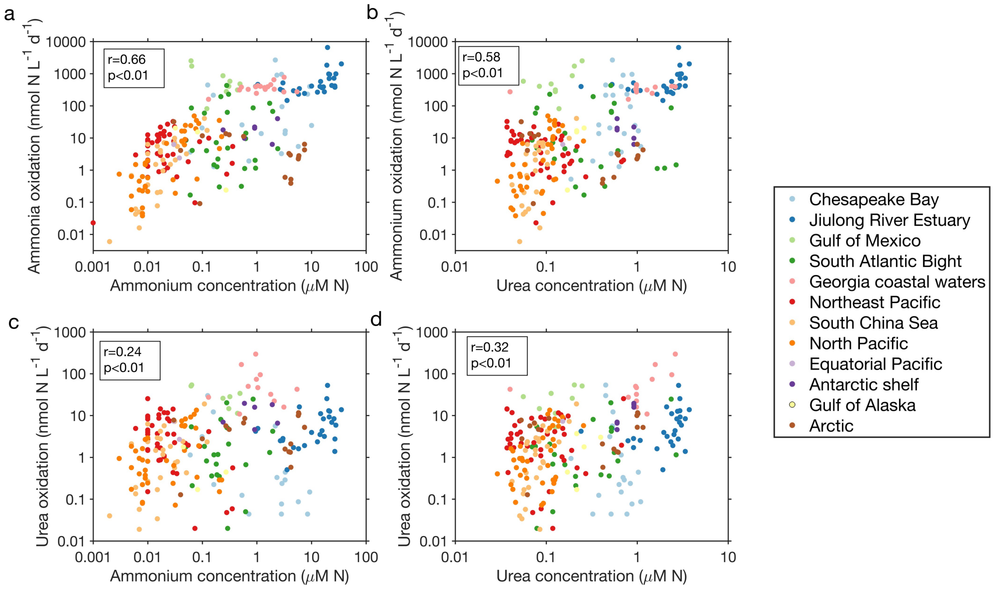

Figure S6. The relationship between substrate concentrations and nitrification rates. Ammonium oxidation vs ammonium (a) and urea concentrations (b); urea oxidation vs ammonium (c) and urea concentrations (d). Data show urea-N concentrations.

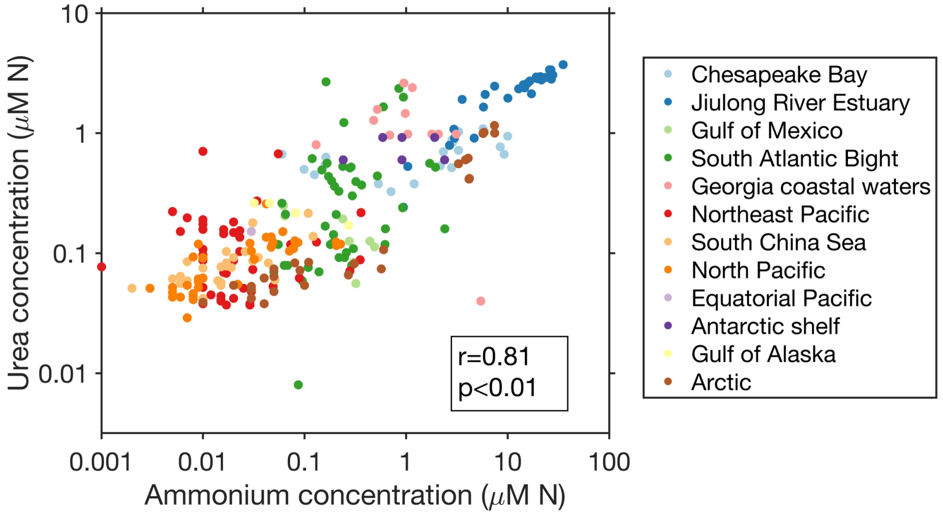

Figure S7. The relationship between urea concentrations and ammonium concentrations in the global ocean. A similar figure was previously shown in Wan et al. (2024), with separated ocean regions.

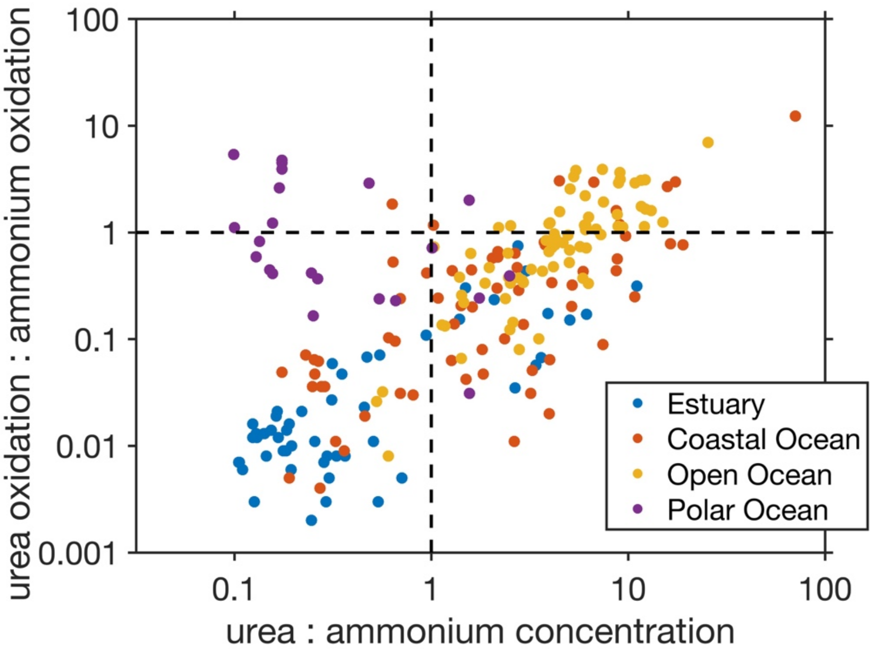

Figure S8. The relationship between the ratio of urea oxidation to ammonium oxidation and the ratio of urea to ammonium concentrations across different marine environments.

a

b

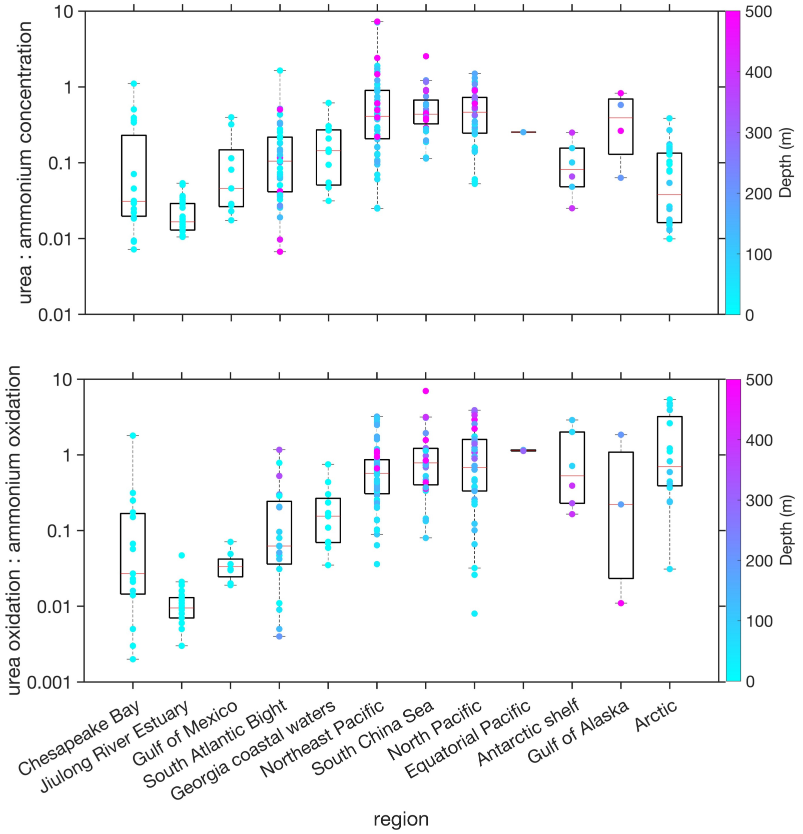

Figure S9. (a) Ratio of urea to ammonium concentrations, and (b) ratio of urea oxidation to ammonium oxidation across different ocean regions. Points are color-coded by the sampling depths. For the boxplots, the red line in each box is the median. The bottom and top of each box are the 25th and 75th percentiles of the observations, respectively. The error bars represent 1.5 times the interquartile range away from the bottom or top of the box.

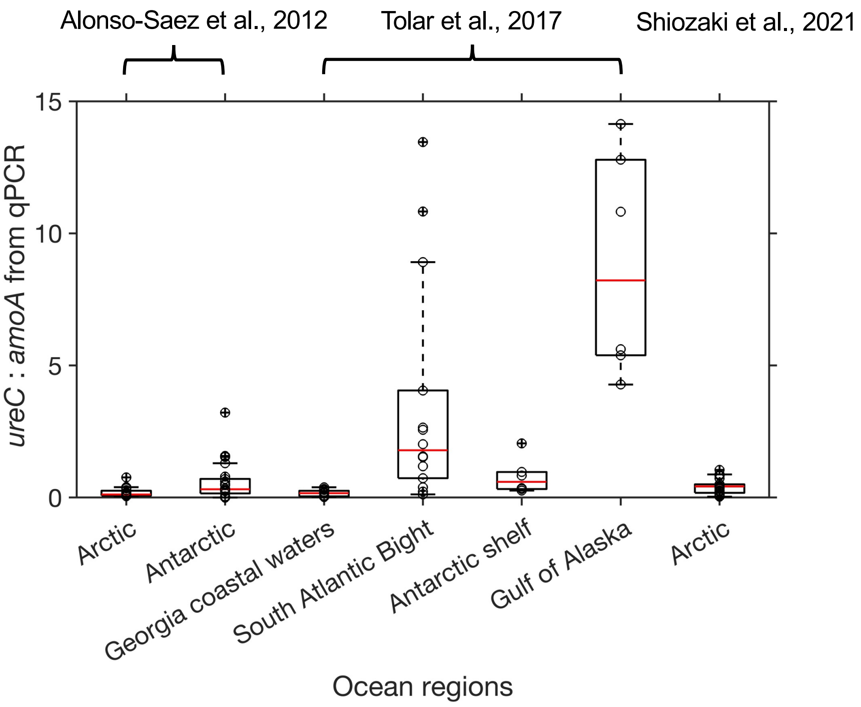

Figure S10. The ratio of *ureC* to *amoA* gene abundance in *Nitrososphaerota* quantified by qPCR in different regions. For the boxplots, the red line in each box is the median. The bottom and top of each box are the 25th and 75th percentiles of the observations, respectively. The error bars represent 1.5 times the interquartile range away from the bottom or top of the box, with red + signs showing outliers beyond that range. Individual data points are shown as circles. Data are extracted from Alonso-Saez et al. (2012), Tolar et al. (2017), and Shiozaki et al. (2021).

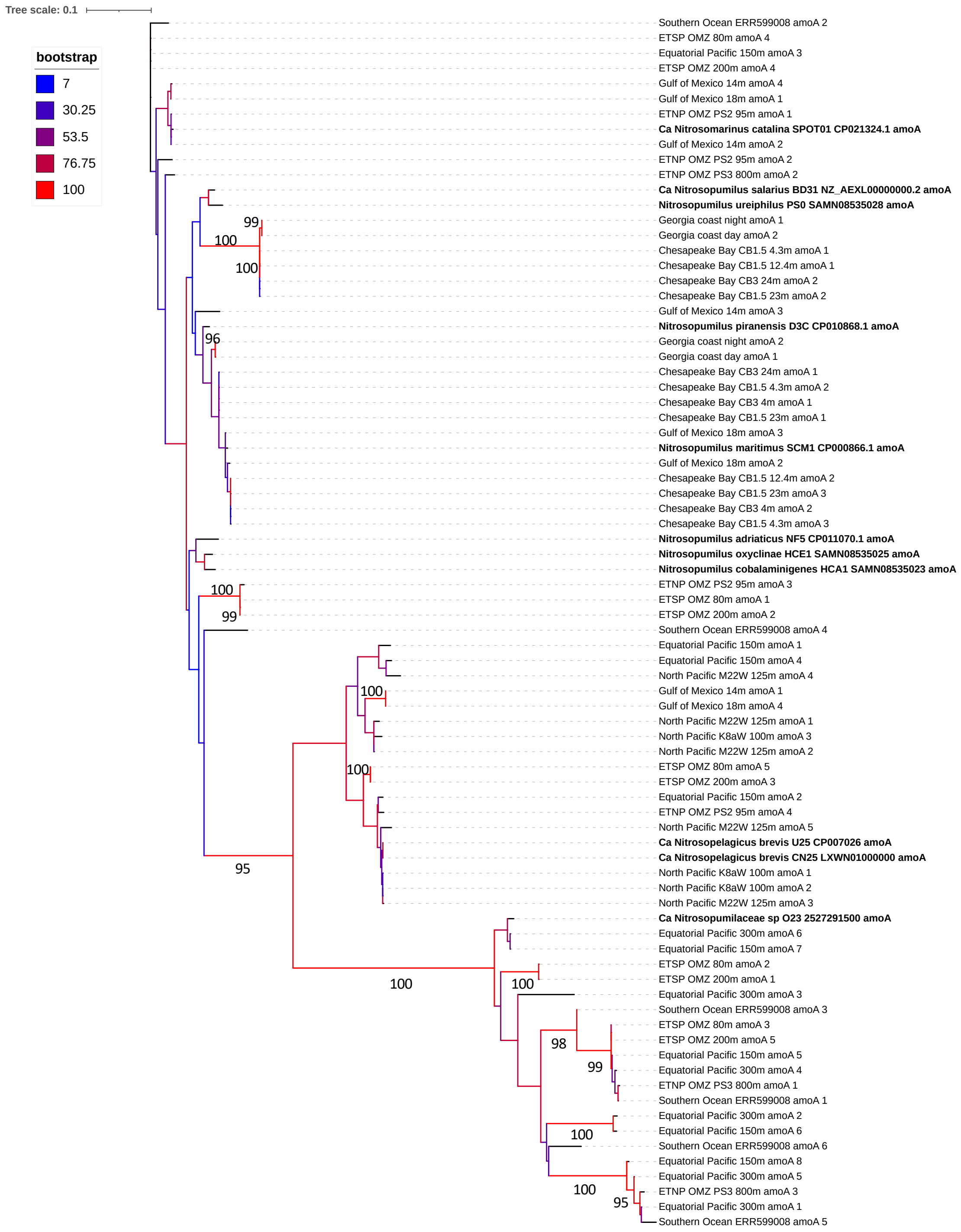

Figure S11. Phylogenetic tree of ammonia-oxidizing archaea *amoA* gene sequences. Reference *amoA* gene sequences (bold) extracted from a few known marine ammonia-oxidizing archaea genomes are used for comparison. Method used to construct the phylogenetic tree is described in the method section. Tree shown is a consensus tree from 100 bootstraps; node color indicates bootstrap confidence and ≥95% confidence is shown near the corresponding branch. Tree scale is in nucleotide substitutions per site.

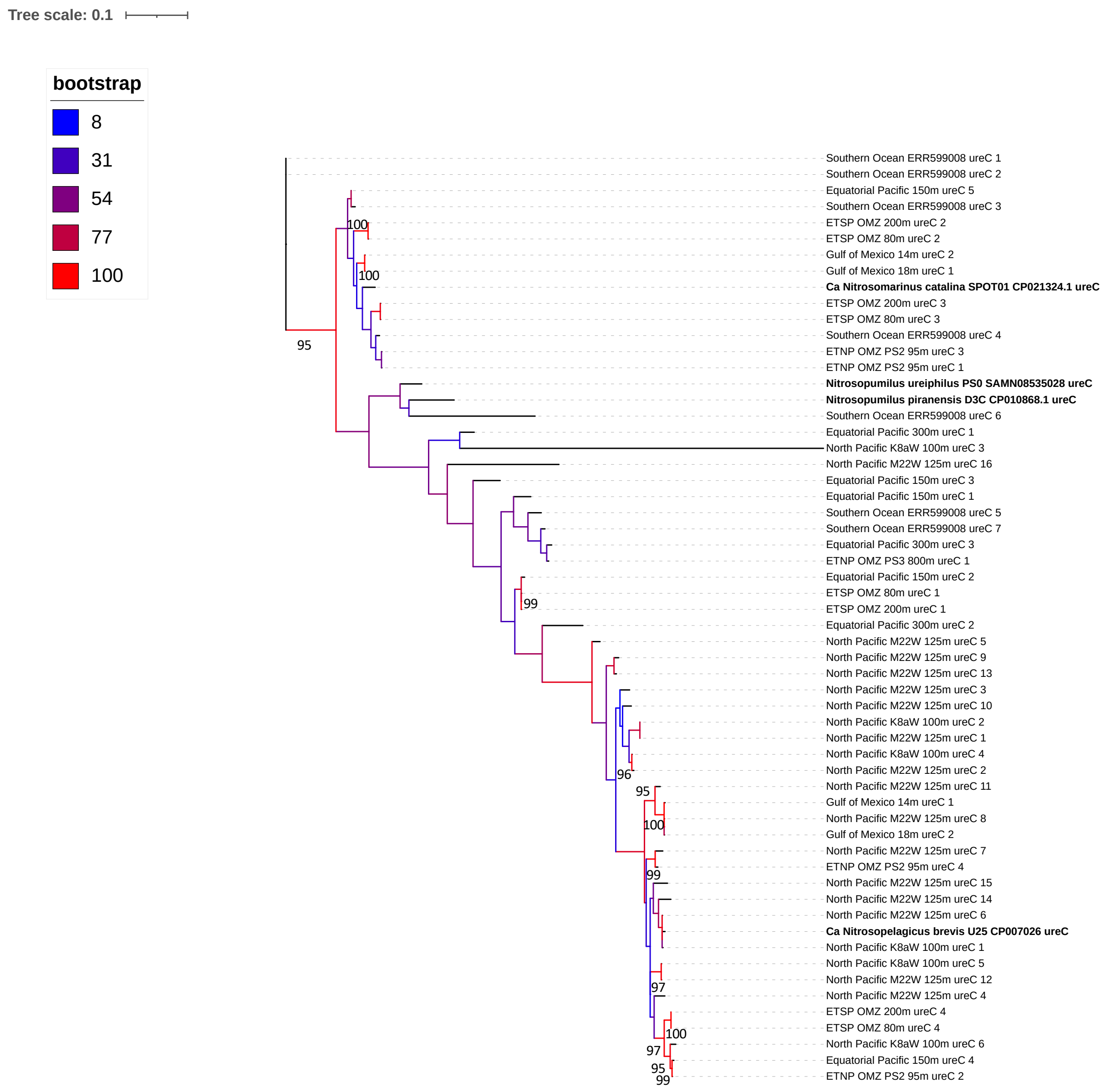

Figure S12. Phylogenetic tree of ammonia-oxidizing archaea UreC amino acid sequences. Reference UreC amino acid sequences (bold) extracted from a few known marine ammonia-oxidizing archaea genomes are used for comparison. Method used to construct the phylogenetic tree is described in the method section. Tree shown is a consensus tree from 100 bootstraps; node color indicates bootstrap confidence and ≥95% confidence is shown near the corresponding branch. Tree scale is in amino acid substitutions per site.

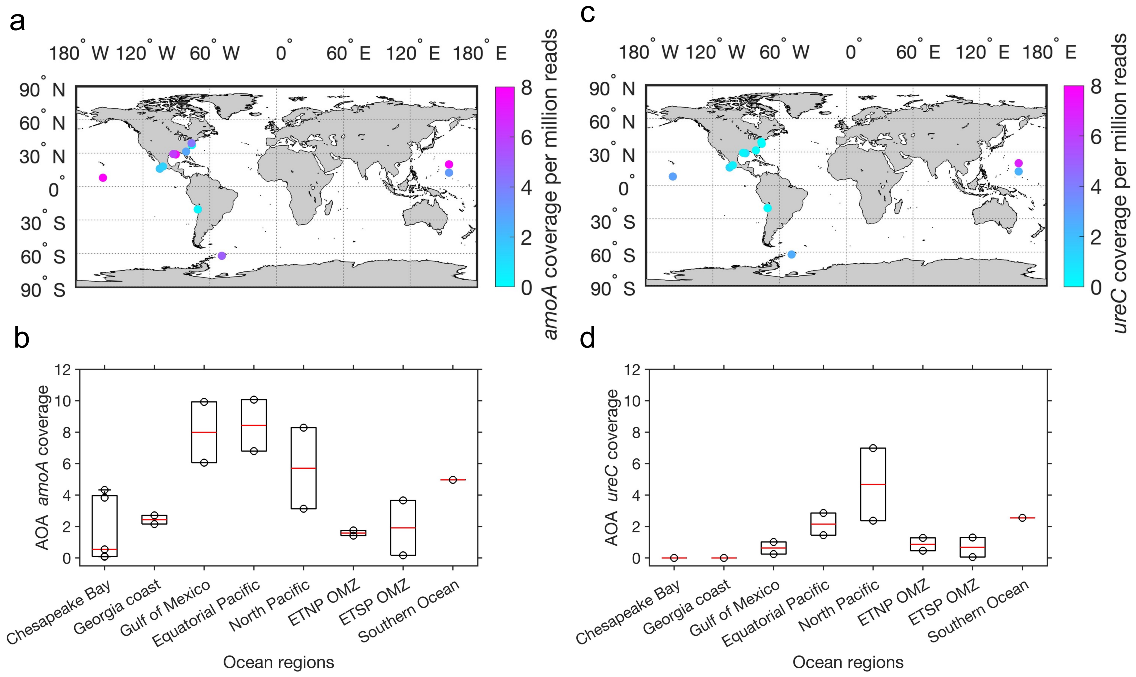

Figure S13. The coverages of *amoA* (a, b) and *ureC* (c, d) in ammonia-oxidizing archaea in metagenomic samples collected from different ocean regions.

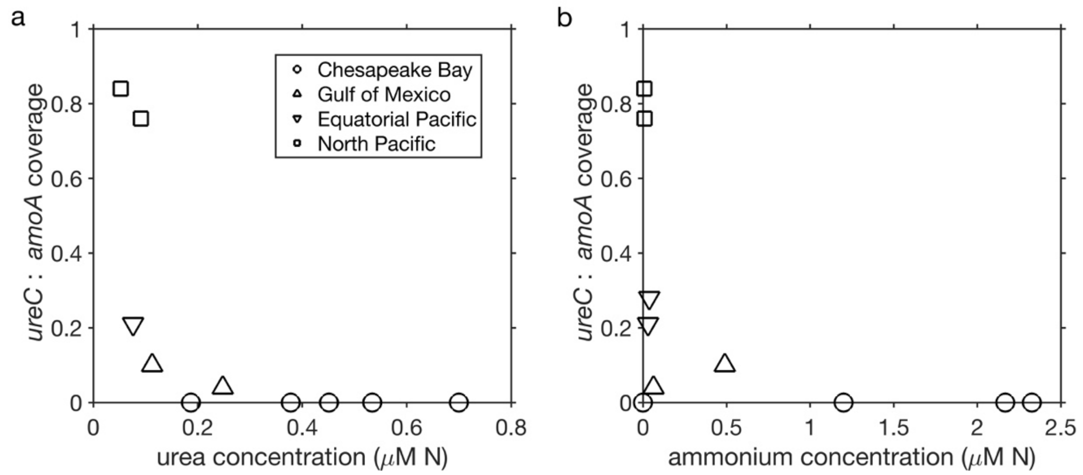

Figure S14. The dependence of ammonia-oxidizing archaea *ureC* : *amoA* ratios on urea (a) and ammonium (b) concentrations across different ocean regions.

References

Alonso-Saez, L., Waller, A.S., Mende, D.R., Bakker, K., Farnelid, H., Yager, P.L. et al. (2012) Role for urea in nitrification by polar marine Archaea. *Proc Natl Acad Sci* **109**: 17989-17994.

Damashek, J., Edwardson, C.F., Tolar, B.B., Gifford, S.M., Moran, M.A., and Hollibaugh, J.T. (2019) Coastal Ocean Metagenomes and Curated Metagenome-Assembled Genomes from Marsh Landing, Sapelo Island (Georgia, USA). *Microbiol Resour Announc* **8**: 10-1128.

Fortin, S.G., Sun, X., Jayakumar, A., and Ward, B.B. (2024) Nitrite-oxidizing bacteria adapted to low oxygen conditions dominate nitrite oxidation in marine oxygen minimum zones. *The ISME Journal* **18**: wrae160.

Kitzinger, K., Padilla, C.C., Marchant, H.K., Hach, P.F., Herbold, C.W., Kidane, A.T. et al. (2018) Cyanate and urea are substrates for nitrification by Thaumarchaeota in the marine environment. *Nature Microbiology* **4**: 234-243.

Salazar, G., Paoli, L., Alberti, A., Huerta-Cepas, J., Ruscheweyh, H.J., Cuenca, M. et al. (2019) Gene Expression Changes and Community Turnover Differentially Shape the Global Ocean Metatranscriptome. *Cell* **179**: 1068-1083 e1021.

Santoro, A.E., Saito, M.A., Goepfert, T.J., Lamborg, C.H., Dupont, C.L., and DiTullio, G.R. (2017) Thaumarchaeal ecotype distributions across the equatorial Pacific Ocean and their potential roles in nitrification and sinking flux attenuation. *Limnology and Oceanography* **62**: 1984-2003.

Shiozaki, T., Hashihama, F., Endo, H., Ijichi, M., Takeda, N., Makabe, A. et al. (2021) Assimilation and oxidation of urea-derived nitrogen in the summer Arctic Ocean. *Limnology and Oceanography* **66**: 4159-4170.

Sun, X., Kop, L.F.M., Lau, M.C.Y., Frank, J., Jayakumar, A., Lücker, S., and Ward, B.B. (2019) Uncultured Nitrospina-like species are major nitrite oxidizing bacteria in oxygen minimum zones. *The ISME Journal* **13**: 2391-2402

Tang, W., Jayakumar, A., Sun, X., Tracey, J.C., Carroll, J., Wallace, E. et al. (2022) Nitrous Oxide Consumption in Oxygenated and Anoxic Estuarine Waters. *Geophysical Research Letters* **49**: e2022GL100657.

Tolar, B.B., Wallsgrove, N.J., Popp, B.N., and Hollibaugh, J.T. (2017) Oxidation of urea-derived nitrogen by thaumarchaeota-dominated marine nitrifying communities. *Environ Microbiol* **19**: 4838-4850.

Wan, X.S., Sheng, H.X., Shen, H., Zou, W., Tang, J.M., Qin, W. et al. (2024) Significance of Urea in Sustaining Nitrite Production by Ammonia Oxidizers in the Oligotrophic Ocean. *Global Biogeochemical Cycles* **38**: e2023GB007996.
